# Interactions among Lacosamide and Second-Generation Antiepileptic Drugs in the Tonic-Clonic Seizure Model in Mice

**DOI:** 10.3390/ijms22115537

**Published:** 2021-05-24

**Authors:** Katarzyna Załuska-Ogryzek, Paweł Marzęda, Paula Wróblewska-Łuczka, Magdalena Florek-Łuszczki, Zbigniew Plewa, Hubert Bojar, Dorota Zolkowska, Jarogniew J. Łuszczki

**Affiliations:** 1 Department of Pathophysiology, Medical University of Lublin, 20-090 Lublin, Poland; katarzyna.zaluska-ogryzek@umlub.pl (K.Z.-O.); pawel.marzeda@umlub.pl (P.M.); paula.wroblewska-luczka@umlub.pl (P.W.-Ł.); 2 Department of Medical Anthropology, Institute of Rural Health, 20-090 Lublin, Poland; magdalena.florek@wp.pl; 3 Department of General, Oncological and Minimally Invasive Surgery, 1st Military Clinical Hospital, 20-048 Lublin, Poland; plewa_z@o2.pl; 4 Department of Toxicology and Food Safety, Institute of Rural Health, 20-090 Lublin, Poland; bojar.hubert@imw.lublin.pl; 5 Department of Neurology, UC Davis School of Medicine, Sacramento, CA 95816, USA; dzolkowska@gmail.com; 6 Isobolographic Analysis Laboratory, Institute of Rural Health, 20-090 Lublin, Poland

**Keywords:** drug–drug interaction, antiseizure medication, maximal electroshock-induced seizures, isobolographic analysis, lacosamide, polygonogram

## Abstract

Combination therapy with two or three antiseizure medications (ASMs) is sometimes a preferred method of treatment in epilepsy patients. (1) Background: To detect the most beneficial combination among three ASMs, a screen test evaluating in vivo interactions with respect to their anticonvulsant properties, was conducted on albino Swiss mice; (2) Methods: Classification of interactions among lacosamide (LCM) and selected second-generation ASMs (lamotrigine (LTG), pregabalin (PGB), oxcarbazepine (OXC), and topiramate (TPM)) was based on the isobolographic analysis in the mouse maximal electroshock-induced seizure (MES) model. Interactions among LCM and second-generation ASMs were visualized using a polygonogram; (3) Results: In the mouse MES model, synergy was observed for the combinations of LCM + TPM + PGB and LCM + OXC + PGB. Additivity was reported for the other combinations tested i.e., LCM + LTG + TPM, LCM + LTG + PGB, LCM + LTG + OXC, and LCM + OXC + TPM in this seizure model. No adverse effects associated with triple ASM combinations, containing LCM and second-generation ASMs were observed in mice; (4) Conclusions: The combination of LCM + TPM + PGB was the most beneficial combination among the tested in this study, offering synergistic suppression of tonic-clonic seizures in mice subjected to the MES model. Both the isobolographic analysis and polygonogram method can be recommended for experimental epileptology when classifying interactions among the ASMs.

## 1. Introduction

Patients with epilepsy require efficacious treatment with current frontline antiseizure medications (ASMs). Approximately 70% of epilepsy patients are sufficiently treated with one drug, but the rest of the patients need polytherapy with two or three ASMs [1]. For these patients, physicians try to combine various ASMs so as to provide them with a significant reduction of seizure activity and/or seizure frequency [2]. Despite the progress in clinical epileptology and several novel ASMs licensed recently for the treatment of epilepsy (i.e., perampanel, cenobamate, and ganaxolone) [3,4,5,6], there is still no clear definition of refractory epilepsy [7,8]. Nevertheless, patients with refractory epilepsy need effective polytherapy with ASMs, but each polytherapy is usually associated with interactions among drugs, whose nature may be pharmacodynamic, pharmacokinetic, or mixed [9]. Although physicians can prescribe their patients more than 25 ASMs, they still have no unanimous recommendations on which of these drugs preferentially combine to offer the epileptic patients the best treatment options [10].

At present, several dual and triple combinations of ASMs have gained clinical approval as effective combinations in the treatment of epilepsy [11,12,13]. However, information about the effective combinations of ASMs has been obtained from some review papers, but there are still no clinical trials evaluating the efficacy of some dual and triple combinations of ASMs. In clinical conditions, it is difficult to directly evaluate the efficacy of the ASMs in various combinations due to a huge number of possible dual and triple combinations of ASMs, the diverse clinical manifestation of seizures, the duration of epilepsy, age of the patients, etc. To help physicians select the proper combinations of ASMs, researchers can test various combinations in preclinical studies and create a ranking list of the most beneficial combinations of ASMs. From a preclinical viewpoint, the best option is to combine ASMs with different molecular targets because the drugs affecting only one target or the same targets may compete with one another and the final effect can be lower than expected [14]. Multi-targeted drugs when combined together can work independently in terms of suppressing seizures, and the drugs in a mixture can be applied in reduced doses, which may contribute to the reduction of the drugs’ toxicity [15]. Both maximizing efficacy and minimizing toxicity are the desirable properties of the favorable combinations that can be clinically recommended [16].

To date, several dual and triple combinations of ASMs have been tested in preclinical studies using the maximal electroshock-induced seizure (MES) test, which is thought to be a model of generalized tonic-clonic seizures in mice [17]. Although various ASMs can be theoretically combined together, taking into account their various molecular mechanisms of action, only experimental verification can assess the in vivo efficacy of ASMs in animals. From a theoretical viewpoint, a combination is efficacious (beneficial) if the drugs comprising the combination produce synergistic interaction [18]. In contrast, the least favorable combination is observed if the drugs produce antagonistic interaction. Although the drugs are theoretically selected to constitute beneficial combinations, only the preclinical testing can unequivocally verify these rationally selected drug–drug combinations, confirming finally the exact type of interactions occurring in vivo between the tested drugs [19]. Of course, it is not guaranteed that combinations of ASMs that synergistically work in mice will also work in humans.

In this study, we aimed to classify the interactions for three-drug combinations among 5 ASMs (namely, lacosamide (LCM), lamotrigine (LTG), oxcarbazepine (OXC), pregabalin (PGB), and topiramate (TPM)), so as to select the most beneficial combinations of ASMs, offering synergistic suppression of tonic-clonic seizures in the mouse MES model by means of the isobolographic analysis of interactions accompanied with a polygonogram method. Both, the isobolographic analysis and polygonogram method are applied by researchers to properly classify and visualize interactions occurring among the studied drugs. Understanding which combinations of ASMs are favorable will be essential for selecting the novel therapies for the patients with refractory epilepsy [20]. Of note, the studied 5 ASMs (LCM, LTG, OXC, PGB, and TPM) are effective in suppressing tonic-clonic seizures in both preclinical in vivo studies in mice [17] and clinical settings in humans [21].

## 2. Results

### 2.1. Anticonvulsant Effects of ASMs When Administered Separately in the Mouse Model of Tonic-Clonic Seizures

All the studied ASMs exerted anticonvulsant effects in the mouse MES model. The log-probit dose–response relationship lines for the tested ASMs were verified in the test for parallelism. All the tested ASMs had their dose–response lines collateral to each other (Supplementary Figure S1). The experimentally derived ED_50_ values for the ASMs, when injected separately, were calculated from linear log-probit equations (Supplementary Figure S1).

### 2.2. Isobolographic Analysis of Interactions among the Studied ASMs in the Mouse MES Model

The three-drug mixtures for various triple combinations tested in this study, produced in a dose-dependent manner, the antiseizure effects in the mouse MES model. The experimentally derived ED_50 exp_ values for various triple combinations of ASMs are presented in Table 1.

**Figure 1:**
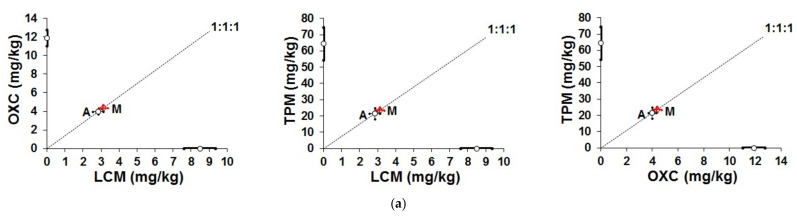

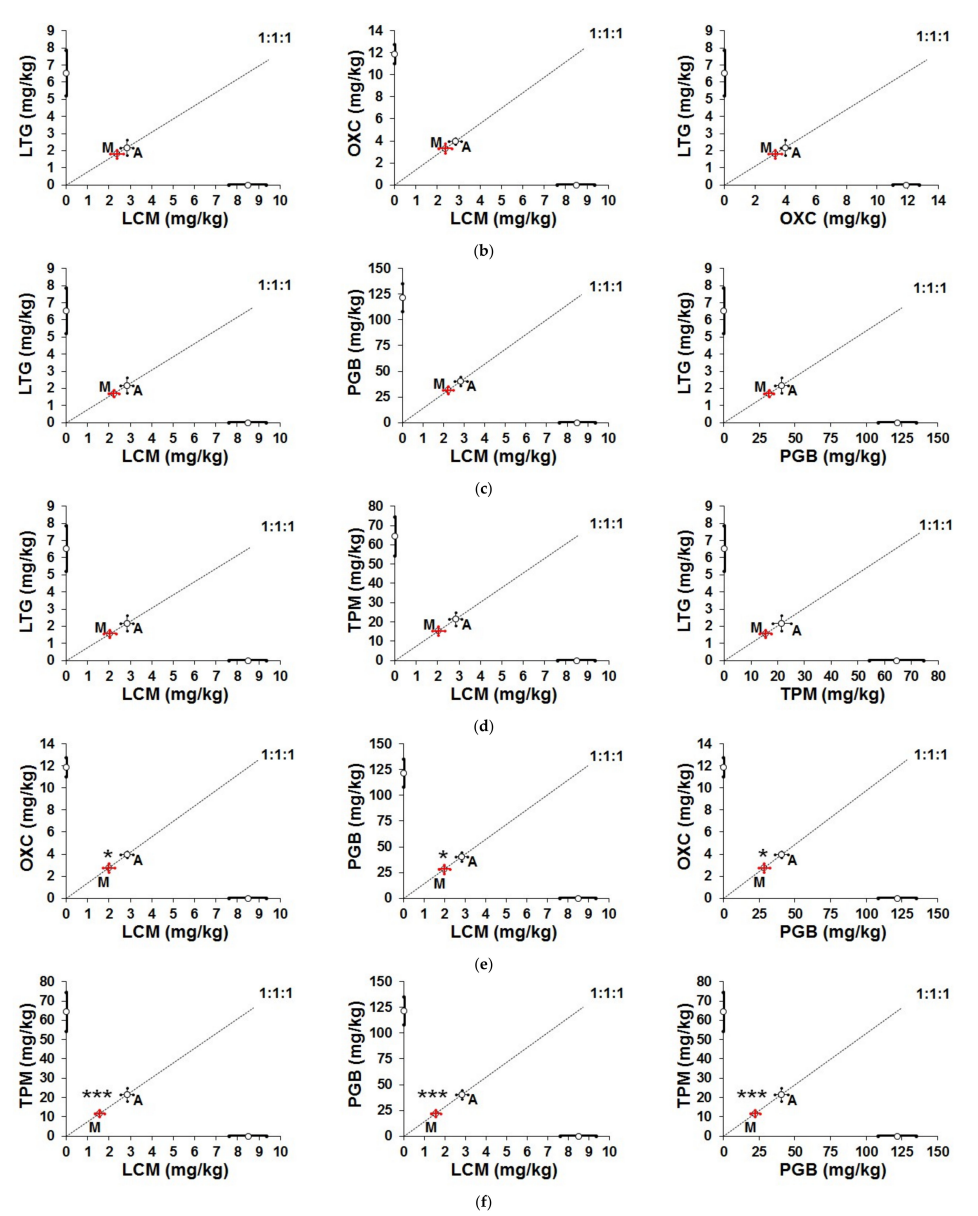
Isobolographic analysis of interactions of various triple ASM combinations: (**a**) additive interaction for the combination of LCM + OXC + TPM; (**b**) additive interaction for the combination of LCM + LTG + OXC; (**c**) additive interaction for the combination of LCM + LTG + PGB; (**d**) additive interaction for the combination of LCM + LTG + TPM; (**e**) synergistic interaction for the combination of LCM + OXC + PGB (* *p* < 0.05—unpaired Student’s *t*-test); (**f**) synergistic interaction for the combination of LCM + TPM + PGB (*** *p* < 0.001—unpaired Student’s *t*-test). On each graph, point A represents the theoretically calculated additive ED_50 add_ value (±S.E.M. as the error bars), whereas point M displays the experimentally-derived ED_50 exp_ value (±S.E.M. as the error bars).

To display the characteristics of interactions among the tested ASMs, the isobolograms were plotted graphically in the Cartesian plot system. Statistical comparison of ED_50 exp_ values with ED_50 add_ values revealed that for the combinations of LCM + OXC + TPM and LCM + TPM + PGB, the difference reached significance (at *p* < 0.05 and *p* < 0.001, respectively), and the observed interaction was synergistic in nature (Table 1; Figure 1). For other combinations of ASMs tested, statistical analysis revealed no significance, therefore, the observed interactions were additive in this tonic-clonic seizure model in mice (Table 1 and Figure 1).

**Figure 2:**
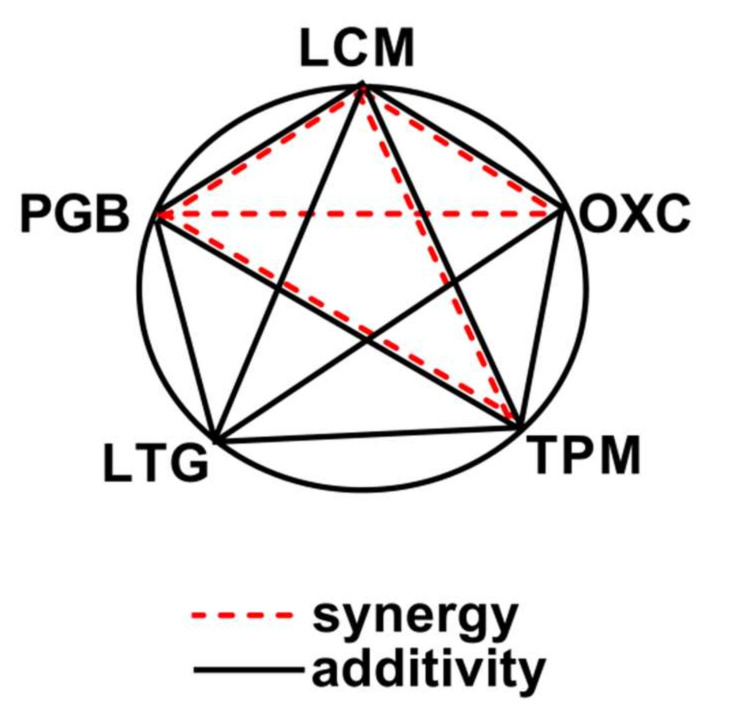
Polygonogram for triple ASM combinations.

To visualize all the observed interactions for various three-drug combinations among ASMs in this study, a polygonogram was drawn (Figure 2).

### 2.3. Interaction Indices for the Tested Triple Combinations of ASMs

To determine the potency for the observed interactions, the interaction index values were calculated as a ratio of the respective ED_50 exp_ and ED_50 add_ values. The lowest interaction index of 0.55 was determined for the combination of LCM + TPM + PGB (Table 2). In contrast, the highest interaction index value of 1.10 was calculated for the combination of LCM + OXC + TPM (Table 2). The interaction indices allowed for the descending arrangement of the triple ASM combinations (Table 2).

### 2.4. Adverse Effect Potentials of ASMs in Combination in the Rotarod and Grip-Strength Tests in Mice

All the studied ASMs in three-drug combinations did not significantly impair motor coordination in animals challenged with the rotarod test. In this case, the balancing time for animals from the control group did not differ from that for animals receiving the combinations of ASM in doses from the mouse MES model (Table 3). Similarly, the combinations of ASMs did not affect skeletal muscular strength in mice subjected to the grip-strength test. The mean strengths of animals from the control group did not differ considerably from those for the mice receiving ASMs in various triple combinations (Table 3).

## 3. Discussion

Experimental assessment of the anticonvulsant action of three-drug mixtures comprising various combinations of second- and third-generation ASM in preclinical conditions allowed us to select the best combinations with the most beneficial antiseizure action in the mouse MES model. The most favorable triple ASM combination in this study was that for LCM + TPM + PGB, which offered the synergistic anticonvulsant effects with an interaction index value of 0.55. Synergistic interaction among the tested ASMs was also observed for the combination of LCM + PGB + OXC in the mouse MES model with an interaction index value amounting to 0.7. The other combinations tested in this study were additive with a tendency towards synergy (i.e., LCM + PGB + LTG, LCM + OXC + LTG, and LCM + LTG + TPM) or additive (LCM + OXC + TPM), in this seizure model.

In clinical conditions, polytherapy with ASMs is prescribed usually for patients with refractory epilepsy. If a patient takes a polytherapy with two or three drugs in combination, all the drugs are administered in fully effective doses each. Thus, the patient takes two or three effective drugs in doses providing separately a complete protection from seizures [31]. In the case of the isobolographic analysis, the combined two or three drugs are administered in reduced doses, which are equivalent to one drug used separately [32,33]. The isobolographic principle statistically compares doses of drugs in mixture, which experimentally protected the mice from seizures (ED_50 exp_), with doses that are theoretically predicted to be additive (ED_50 add_). Calculation of these additive values for various drug mixtures is based on the equation of mass-action law, whose final effect is always equal to one drug [32,33]. According to this equation, doses of drugs in mixture were in a fixed drug dose ratio of 1:1:1 and were substantially reduced, reflecting one ED_50_ value of the ASMs.

Synergistic interactions observed isobolographically in preclinical conditions are always associated with reduced drug doses, which are not expected in clinical settings with triple therapy with ASMs [34,35]. We are fully aware of the fact that doses of ASM used in preclinical studies cannot be directly extrapolated to clinical conditions. At present, there is an algorithm allowing for the calculation of the proper dosages of drugs when translating experiments from animals to humans [36]. Generally, the antiseizure effect produced by the combination of ASMs results from the interaction among the ASMs and the activation of their different molecular mechanisms of action. It is expected that the same molecular mechanisms are responsible for the observed interaction in both animal and human studies. On the other hand, clinicians should avoid combining ASMs that produce antagonistic interactions in the mouse MES model. Previously, it has been confirmed that combinations exerting antagonistic interactions in preclinical studies also produced unfavorable effects in epilepsy patients. The best examples documenting antagonistic interactions in both preclinical and clinical studies are those for the combinations of LTG with OXC [18] or LTG with CBZ [37], in which the combined effects of LTG with CBZ or OXC were lower than each of the drugs used separately [38,39,40].

The isobolographic analysis of interaction was predicted to determine pharmacodynamic interactions occurring among the tested drugs. Additionally, to visualize the observed interactions among ASMs, we used the polygonogram method. Although the polygonogram method in the visualization of interactions has been known since 2000 [41], its application in experimental epileptology started two years ago [42]. Of note, the isobologram displays interactions for only one combination of drugs, whereas a polygonogram illustrates interactions for several drugs used in various drug–drug combinations. A polygonogram allows us to quickly assess which of the tested ASM combinations are better than others, with respect to the protection from seizures. This is the reason for presenting the results of this study as a polygonogram and to recommend its application as an obligatory method in this type of research. Of note, researchers using the polygonogram method are forced to perform several experiments in a precisely defined period of time, contributing evidently to the reduction in the number of animals used, which is in agreement with the “3Rs” rule (Replacement, Refinement, Reduction) and ARRIVE guidelines when conducting experiments on animals [43]. This method reduces the total number of laboratory animals used in experimental conditions by using the same available results several times for various calculations (i.e., ED_50_ values for ASMs when administered separately). The polygonogram provides also information on several combinations tested in the same experimental conditions [41].

Although several animal models of generalized tonic-clonic seizures are available in preclinical studies [44], the most frequently used model is the MES model in mice [45,46]. Other models of generalized tonic-clonic seizures in mice (i.e., pilocarpine-; N-methyl-D,L-aspartate-; kainic acid-induced seizures) are specific models of chemically-induced seizures, whose usage is limited and requires some special conditions [47,48]. The MES model in mice is easy to reproduce, and each animal subjected to this model develops seizure activity. In contrast, other models are not only time-consuming but also expensive and cost-ineffective, especially, if experiments on animals are performed according to the established protocols [47,48]. Additionally, the MES model in mice is recommended as a screen test for selecting various naturally occurring and newly synthesized compounds with anticonvulsant in vivo properties against tonic-clonic seizures [49,50]. In this test, ASMs and their combinations were screened to choose the most effective treatment options that offer suppression of tonic-clonic seizures. This is the main reason to test various three-drug combinations of ASMs in the mouse MES model. On the other hand, in experimental epileptology, there are several animal seizure models reflecting various types of seizures that occur in humans. For instance, pentylenetetrazole-induced seizures reproduce myoclonic seizures and nonconvulsive absence epilepsy in humans; 6-Hz stimulation induced seizures mimic limbic refractory epilepsy in humans; the MES model is thought to be an animal model of generalized tonic-clonic seizures and, to a certain extent, of partial epilepsy in humans [46,47,51]. Undoubtedly, the diversity of experimental in vivo models used in preclinical studies affects the evaluation of the anticonvulsant efficacy of some ASMs in combination. Previously, it has been reported that the same combination of two drugs exerted different interactions in various experimental models of epilepsy. More specifically, WIN-55,212-2 mesylate (a non-selective cannabinoid CB1 and CB2 receptor agonist) when combined with phenobarbital, synergistically potentiated the anticonvulsant action of the latter drug in the 6-Hz stimulation induced seizure model in mice [52]. Simultaneously, WIN 55,212-2 mesylate exerted additive interaction with phenobarbital in both the MES and pentylenetetrazole-induced seizure models in mice [53,54]. Similarly, WIN 55,212-2 mesylate synergistically potentiated the antiseizure action of valproate in both, 6-Hz stimulation and MES models, but it was additive when combined with valproate in the pentylenetetrazole-induced seizure model in mice [52,53,54]. The diversity of interactions observed between WIN 55,212-2 mesylate and two classical ASMs in various experimental models of epilepsy may testify about the model-specific interactions that may occur when combining the ASMs.

Notably, in this study, the interaction index was used as a measure of the potency of interactions for ASM combinations. The lower the interaction index value, the greater synergy is observed in experimental conditions. In this study, statistical analysis of data provided only information on whether or not the analyzed values significantly differed from each other. Even if two values are significantly lower than the theoretically calculated additive values, we cannot precisely indicate which of the tested combinations is better (stronger) than others. In this analysis, the interaction index is the best predictor for characterizing the potency of interactions among ASMs [55,56]. Due to the interaction index values, we can classify interactions more precisely. On the other hand, we are aware of the fact that the classification of interaction based exclusively on border values for synergy, additivity, and antagonism is not precise and without a statistical test it cannot be used alone for the classification of interactions [56,57]. Presently, the application of interaction index values when assessing the potency of interactions among the tested drugs, after confirming the existence of significant difference with one of the commonly used statistical tests (i.e., the unpaired Student’s *t*-test), should be recommended in this type of research.

In this study, all pharmacodynamic interactions among newer ASMs were also evaluated with respect to the drugs’ propensities to produce side effects that could be potentially harmful to patients in clinical settings. It is noteworthy that doses of ASMs used in triple combinations reflected those doses that protected the animals from seizures. In this study, we assessed the animals’ behavior in the grip-strength and rotarod tests. Both, the rotarod and grip-strength tests are commonly used as screen tests in experimental epileptology when evaluating the influence of the tested drugs on animals’ behavior, especially, when testing drugs affecting CNS [55,58,59]. It is important to note that some of the second- and third-generation ASMs used in this study (i.e., PGB and TPM) produce antinociceptive effects in experimental animals and are used clinically to treat neuropathic pain in suffering patients [60,61,62,63,64]. Additionally, LTG, OXC, and LCM can be used as off-label drugs to treat neuropathic pain, trigeminal neuralgia, and other pain ailments due to their antinociceptive properties [65,66]. Therefore, we did not assess the effects of ASMs in combinations on long-term memory processes in mice in the step-through passive avoidance task because this test is based exclusively on nociception produced by an aversive stimulus during the testing procedure and thus, the animals do not experience the aversive stimulus when receiving antinociceptive ASMs. In other words, the observed effects in this test would falsely indicate impaired long-term memory in the mice due to the antinociception exerted by ASMs during the aversive stimulation. The problem of the evaluation of nociceptive reactions in animals receiving ASMs and subjection to the passive avoidance task has been discussed previously [67,68]. Evaluation of active learning and memory processes in mice receiving three various ASMs and being subjected to the Y-maze or Morris Water Maze tests needs additional experimental groups of animals to be tested so as to determine the effects of ASMs when used separately and in dual combinations (for more information see: [68,69,70]). On the other hand, evaluation of the pain threshold in animals receiving the ASMs in combinations and challenged with the step-through passive avoidance task needs also experimental animals [67]. Being aware of the restrictions related with the “3Rs” rule and ARRIVE guidelines [43], when conducting experiments on animals, the evaluation of learning and memory processes in mice was not performed in this study.

Results from the rotarod test indicated that none of the studied ASM combinations considerably altered motor coordination in experimental animals because no significant changes in the animals’ balance and coordination were reported in the mice. Additionally, results from the grip-strength test reported that the ASM combinations did not impair skeletal muscular strength in animals. Thus, a lack of any acute adverse effects in animals in the rotarod and grip-strength tests confirmed that the ASM combinations were free of any potential side effects. Of note, both behavioral tests used in this study were sensitive enough to detect any subtle changes in experimental animals. As it was documented earlier, the grip-strength test allowed to detect doses of ASM that reduced muscular strength in the mice [58]. Moreover, in the grip-strength test, it was possible to detect drugs that raised the muscular strength in mice (i.e., sildenafil) [71]. The rotarod test also allowed for the determination of doses of ASM that impaired motor coordination and disturbed balance in animals [72,73]. It is worthwhile mentioning that the evaluation of adverse effects was performed after acute (single) administration of ASM in combination.

It should be clearly stated that the interactions among three drugs were evaluated after single drug dosing. Each mixture of three ASMs was administered singly as three separate injections of ASMs. In clinical settings, the ASMs are usually administered chronically. In this experimental model of epilepsy, the mixtures of three drugs were injected from three different syringes in order not to mix the drugs in one syringe before injection. If the drugs are mixed together in one syringe, a pharmaceutical interaction may occur [9,74]. Of note, pharmaceutical interaction is observed if one of the drugs in the mixture inactivates the other drugs before the mixture is injected into a living organism (i.e., outside an organism—ex vivo). If a pharmaceutical interaction occurs, the mixture produces lower effects than the particular drugs when used singly [57].

To our best knowledge, the combination of LCM + PGB + TPM with an interaction index of 0.55 is the best triple-drug combination observed in preclinical studies in the mouse MES model (Table 2). This combination can be recommended as a treatment option for patients with refractory epilepsy. There is no doubt that we still need combinations of ASMs offering the epileptic patients the effective treatment resulting in a state of seizure freedom. Previously, it has been found that some ASMs in combination produced antagonistic, additive, or synergistic interactions in the mouse MES model (Table 2). Considering the type of interactions occurring in preclinical studies on animals, we can recommend not only the combinations offering synergy in animals, but also those with an additive type of interaction, if the anticonvulsant efficacy of such combinations outweighs the risk of the appearance of adverse effects in epilepsy patients. In this study, it was possible to compare the anticonvulsant action of several triple ASM combinations in the MES model in mice. Due to the interaction index, it was possible to select the most beneficial combinations that could be recommended in clinical settings. In this study, we compared the anticonvulsant properties of triple ASM combinations by arranging them as descending with respect to the observed types of interactions from antagonistic to additive and synergistic (Table 2). All these combinations were tested in preclinical conditions, although the combinations were preferentially selected due to their favorable and theoretically predicted profiles. After conducting experiments on animals, the combinations were finally verified and their profiles were confirmed as synergistic or were classified as additive or antagonistic.

Triple ASM combinations should also be compared to their dual ASM combinations that constitute the tested combinations. In this study, all triple combinations contained LCM as the leading drug added to various dual combinations. Assessment of the anticonvulsant action of dual combinations revealed that PGB combined with LTG, OXC, and TPM exerted additive interaction in the mouse MES model [75]. The combinations of LTG with TPM and OXC with TPM exerted synergistic interaction in the mouse MES model [18,37]. Only the combination of LTG with OXC exerted antagonistic interaction in the MES model in mice [18]. Unfortunately, the combinations of LCM with second- and third-generation ASMs have not been tested in the mouse MES model, so it is impossible to present the interaction profiles for the dual combinations comprising of LCM. Considering the above-mentioned fact, it can be concluded that the addition of LCM (i.e., the drug which selectively potentiates the slow inactivation of voltage-gated sodium channels in active neurons in the epileptic focus [76,77]) to the combination of OXC + TPM changed the interaction type from synergistic (OXC + TPM) to additive (LCM + OXC + TPM). Similarly, the synergistic interaction for the dual combination of LTG + TPM changed to an additive one after adding LCM (LCM + LTG + TPM). In contrast, the antagonistic interaction for OXC + LTG transformed to additive after adding LCM (LCM + OXC + LTG) in the mouse MES model. Of note, the synergy observed for the combinations of LCM + PGB + TPM and LCM + OXC + PGB resulted probably from various multi-targeted mechanisms of action of the examined ASMs in combinations. The slow inactivation of voltage-gated sodium channels evoked by LCM [76], accompanied by the blockade of *α*2*δ* subunits of calcium channels by PGB [78,79], and the OXC-mediated blockade of calcium and sodium channels in neurons [80], evidently contribute to the synergistic interaction in the mouse MES model. Regarding TPM, the drug with its multi-targeted mechanisms of action related to the blockade of sodium channels, activation of specific GABA-A receptor isoforms, inhibition of AMPA/kainate receptors, and selective inhibition of type II and IV forms of carbonic anhydrase [81,82,83,84,85] can also contribute to the synergistic interaction of the combination of LCM + PGB + TPM in the mouse MES model.

It is important to note that in this study, all ASMs in triple combinations were tested experimentally on adult (8-week-old) animals, but we are aware of the fact that the anticonvulsant effects tested on younger and older animals might theoretically differ, due to different pharmacokinetic parameters related with absorption, distribution, metabolism, and elimination of ASMs, while testing the respective three-drug mixtures. Since different ED_50_ values are reported for juvenile and adult mice, the interactions occurring among the ASMs in triple combinations might also vary, but this hypothesis needs to be experimentally proved in additional in vivo studies. On the other hand, molecular mechanisms of the anticonvulsant action of ASMs should not differ significantly with respect to the age of the tested animals, and no significant changes in the types of interactions are expected in juvenile and adult animals, especially, if ASMs belong to the second- and third-generation ASMs.

The main limitation in this study is the lack of measurement of concentrations of ASMs. Unfortunately, pharmacokinetic interactions were not verified because the drugs were injected singly. During the single administration of drugs, the activation and/or inhibition of CYP isoenzymes in the liver is less likely. Thus, pharmacokinetic interaction among the tested drugs is also unlikely. Furthermore, the doses of the three drugs in mixture (reflecting the ED_50 exp_ value) were too low, therefore, they were unable to evoke pharmacokinetic interactions. In our previous studies, based on the isobolographic analysis of interactions, we found no pharmacokinetic interactions between ASMs belonging to the first-, second-, and third-generations of ASMs. Besides, the novel ASMs (licensed and approved for the treatment of epilepsy) are designed and created as the drugs with maximal anticonvulsant activity and minimal toxicity, along with their ideal or nearly ideal pharmacokinetic properties. This is the reason not to measure total brain concentrations of ASMs in this study.

## 4. Materials and Methods

### 4.1. Animals

Adult male CD-1 mice (8-week-old, weighing 20 to 27 g) were used in this study. The animals were kept in standardized laboratory conditions. Each experimental group contains 8 mice. All experiments run in this study complied with the ARRIVE guidelines, the Guide for the Care and Use of Laboratory Animals of the National Institutes of Health, and the EU Directive 2010/63/EU for animal experiments. All protocols were approved by the Local Ethics Committee for Animal Experimentation at the University of Life Sciences in Lublin, Poland. The total number of mice used in this study was 336 (i.e., 280 mice in the MES model and 56 mice in the grip-strength and rotarod tests).

### 4.2. Drugs

LCM (UCB Pharma, Brussels, Belgium), LTG (Glaxo Wellcome, Kent, UK), OXC (Novartis Pharma AG, Basel, Switzerland), PGB (Pfizer Ltd., Sandwich, Kent, UK), and TPM (Cilag AG, Schaffhausen, Switzerland) were suspended in a 1% aqueous solution of Tween 80 (Sigma-Aldrich, Poznan, Poland) in distilled water. All the drugs were administered systemically (ip) in a volume of 5 mL/kg bodyweight. LCM and OXC were injected 30 min, LTG and TPM 60 min, and PGB 120 min before the MES model and behavioral tests, as recommended elsewhere [86,87]. All experiments were conducted blindly by experienced observers.

### 4.3. Maximal Electroshock-Induced Tonic-Clonic Seizures in the Mouse Model

Tonic-clonic seizure activity in mice was evoked by alternating current (50 Hz, 25 mA, 500 V, 0.2 s stimulus duration) delivered from a rodent shocker using auricular electrodes. Doses of the ASMs when used alone and in triple combinations were transformed logarithmically (log to the base 10), while the antiseizure effects produced by the ASMs in the mice were transformed to probits of response, as recommended elsewhere [88]. Subsequently, from linear log-probit equations, the median effective doses (ED_50_ values ±S.E.M.) of the investigated ASMs (that protected 50% of the mice from tonic-clonic seizures) were calculated, as it was described earlier [89]. Similarly, the transformation of increasing doses of the three-drug mixtures for the respective combinations (in a constant ratio combination of 1:1:1) to the logarithms to the base 10, and the antiseizure activity produced by the three-drug combinations from the mouse MES model to probits of response, allowed for the calculation of the experimentally-derived median effective doses (ED_50 exp_ values ±S.E.M.) for the investigated three-drug combinations against tonic-clonic seizures in mice, as described earlier [17,23,24,26,28,30]. The number of mice used for the calculation of 5 ED_50_ values for LCM, LTG, OXC, PGB, and TPM when administered alone was 136. The number of mice used for the calculation of ED_50 exp_ values for 6 various ASM combinations was 144. Thus, in the mouse MES model we used a total of 280 mice.

### 4.4. Isobolographic Analysis of Interactions

Determination of the parallelism of the dose–response relationship lines for ASMs (when used alone) precedes the isobolographic analysis of interaction, as recommended earlier [90]. Subsequently, the interactions for 6 various three-drug combinations in the mouse MES model were classified isobolographically, as described earlier [91]. For this purpose, the median effective additive doses (ED_50 add_ values ±S.E.M.) for three-drug mixtures were calculated from the respective equations of mass-action law, as recommended elsewhere [92]. The ED_50 add_ values are doses of three-drug mixtures, theoretically predicted to protect half of the tested mice from tonic-clonic seizures. Of note, doses of particular ASMs in the mixtures were in the same constant and equal proportion of 1:1:1, as recommended earlier [18,19,30,75,93,94,95,96,97,98,99,100]. The experimentally-derived median effective doses (ED_50 exp_ values ±S.E.M.) for three-drug mixtures in the fixed proportion of 1:1:1 in the mouse MES model were determined from the log-probit linear regression analysis, as suggested earlier [88]. Isobolographic analysis classified drug–drug interactions as supra-additive (synergistic), additive, and sub-additive (antagonistic) [91]. Detailed information on the isobolographic concepts in experimental epileptology has been previously published [89,94,101]. Visualization of all the types of interactions observed for three-drug mixtures in the mouse MES model, was performed by means of polygonogram, as recommended earlier [102,103].

### 4.5. Grip-Strength Test

In the grip-strength test, the effects of 6 various three-drug combinations on skeletal muscular strength in mice were quantified, as recommended elsewhere [58,104]. The mice, after receiving the respective three-drug mixtures, were subjected to the measurement of skeletal muscular strength of their forepaws, as described earlier [105,106,107]. In this test, each mouse was lifted by the tail and placed on the stainless steel grid (8 cm × 8 cm) connected to the electronic transducer. The animal after grabbing the grid with its forepaws was moved back by the experimenter until the mouse released the grip. The maximal force of the animal’s forepaws before releasing the grid was recorded and analyzed as skeletal muscular strength (in milliNewton per gram of bodyweight (mN/g) as means (±S.E.M.) of 8 mice).

### 4.6. Rotarod Test

In the rotarod test, the effects of 6 various three-drug combinations on balance and motor coordination in mice were quantified, as recommended elsewhere [72]. The mice, after receiving the respective three-drug mixtures, were subjected to the measurement of balance and motor coordination, as described earlier [108,109]. In this test, each mouse was placed on the rotating cylinders with a constant speed of 6 rpm and the animal has to move and retain in equilibrium for 120 s. In the rotarod test, the time spent by each animal on the rotating cylinder before falling from the rod was measured and analyzed as the balance time (as median time with 25th and 75th percentiles).

### 4.7. Statistical Analysis

The ED_50_ values for ASMs when used alone and the experimentally-derived ED_50 exp_ values (±S.E.M.) for various triple ASM combinations from the mouse MES model were calculated by log-probit analysis [88]. Statistical comparison of the ED_50 exp_ values with their respective and theoretically predicted to be additive ED_50 add_ values was performed by means of the unpaired Student’s *t*-test, as recommended elsewhere [110]. Statistical comparison of skeletal muscular strengths in animals for the respective ASM combinations was performed with one-way ANOVA, as recommended elsewhere [71]. Statistical comparison of the balance times in animals subjected to the rotarod test was performed with Kruskal-Wallis non-parametric test, as recommended elsewhere [111]. Statistical significance was observed if differences among values were at *p* < 0.05. All statistical calculations were performed by means of the GraphPad Prism software (version 7.0 for Windows; GraphPad Software, San Diego, CA, USA).

## 5. Conclusions

Synergistic interactions for the triple combinations of LCM + PGB + TPM and LCM + OXC + PGB in the mouse MES model could be recommended for further clinical practice, even if they assume only a certain predictive value. Evaluation and characteristics of interactions among the ASMs should always be performed by means of both, isobolographic analysis and polygonogram, providing finally a simple way to visualize the beneficial ASM combinations. Among the sic combinations tested, only two occurred synergistic in the mouse MES model. The remaining ASM combinations exerted additive interactions in this animal seizure model. Preclinical verification, as an intermediate step in evaluating antiseizure medication of ASM combination, is necessary for selecting the best combinations of ASMs.

## Figures and Tables

**Table 1:** Isobolographic analysis of interactions among three ASMs.

Drug Combination	ED_50 exp_	n_exp_	ED_50 add_	n_add_	Unpaired *t*-Test	Interaction
LCM + OXC + TPM	31.06 ± 2.34	24	28.25 ± 3.37	50	t_71.99_ = 0.685; *p* = 0.496	Additivity
LCM + LTG + OXC	7.50 ± 0.98	32	8.97 ± 0.51	42	t_47.43_ = 1.331; *p* = 0.190	Additivity
LCM + LTG + PGB	35.59 ± 3.86	24	45.52 ± 4.46	42	t_62.71_ = 1.684; *p* = 0.097	Additivity
LCM + LTG + TPM	19.06 ± 2.70	24	26.47 ± 3.37	34	t_55.91_ = 1.716; *p* = 0.092	Additivity
LCM + OXC + PGB	33.04 ± 4.62 *	24	47.30 ± 4.45	58	t_63.44_ = 2.223; *p* = 0.030	Synergy
LCM + TPM + PGB	35.50 ± 5.28 ***	24	64.80 ± 5.02	50	t_60.26_ = 4.022; *p* = 0.0002	Synergy

* *p* < 0.05 and *** *p* < 0.001 vs. the respective ED_50 add_ value. n_exp_—total number of animals from experimental groups at those doses, whose anticonvulsant effects ranged from 4 to 6 probits; n_add_—total number of animals at doses predicted to be additive, calculated from the equation of additivity.

**Table 2:** Characteristics of interactions for triple combinations of ASMs.

Three-Drug Combination	Interaction	Interaction Index	Reference
LCM + CBZ + VPA	Antagonism	1.32	[22]
LCM + LTG + VPA	Antagonism	1.27	[23]
LCM + CBZ + PB	Additivity	1.18	[24]
LCM + OXC + TPM	Additivity	1.10	this study
LCM + LTG + PB	Additivity	1.07	[25]
LCM + CBZ + LTG	Additivity	1.05	[26]
PB + LTG + OXC	Additivity	0.94	[17]
LCM + LTG + OXC	Additivity	0.84	this study
CBZ + PB + VPA	Additivity	0.81	[27]
LCM + LTG + PGB	Additivity	0.78	this study
LCM + LTG + TPM	Additivity	0.72	this study
LCM + OXC + PGB	Synergy	0.70	this study
PB + LTG + PGB	Synergy	0.64	[17]
PB + OXC + PGB	Synergy	0.61	[17]
PB + OXC + TPM	Synergy	0.56	[17]
PB + LTG + TPM	Synergy	0.56	[17]
LCM + TPM + PGB	Synergy	0.55	this study
PB + PHT + PGB	Synergy	0.53	[28]
OXC + PGB + TPM	Synergy	0.51	[29]
PB + TPM + PGB	Synergy	0.48	[17]
CBZ + PB + TPM	Synergy	0.46	[30]

CBZ—carbamazepine; LCM—lacosamide; LTG—lamotrigine; OXC—oxcarbazepine; PB—phenobarbital; PGB—pregabalin; PHT—phenytoin; TPM—topiramate; VPA—valproate.

**Table 3:** Potential acute adverse effects in mice subjected to the grip-strength and rotarod tests.

Drug Combination	Muscular Strength (mN/g)	Balancing Time (s)
Vehicle + vehicle + vehicle	39.12 ± 1.69	120 (120; 120)
LCM + OXC + TPM	38.67 ± 1.67	120 (120; 120)
LCM + LTG + OXC	39.27 ± 1.98	120 (120; 120)
LCM + LTG + PGB	38.83 ± 0.93	120 (120; 120)
LCM + LTG + TPM	41.83 ± 1.38	120 (120; 120)
LCM + OXC + PGB	39.43 ± 1.27	120 (120; 120)
LCM + TPM + PGB	41.38 ± 1.50	120 (120; 120)
Statistics	F(6;49) = 0.698; *p* = 0.652	KW = 5.093; *p* = 0.532

Each experimental group consisted of 8 mice. LCM—lacosamide; LTG—lamotrigine; OXC—oxcarbazepine; PGB—pregabalin; TPM—topiramate.

## Data Availability

The data presented in this study are available in the article and Supplementary Material here.

## Supplementary Materials

The following are available online at https://www.mdpi.com/article/10.3390/ijms22115537/s1, Figure S1: Log-probit dose–response relationship lines for ASMs when injected separately.
