# Supplementary figures and images for: Interactions among Lacosamide and Second-Generation Antiepileptic Drugs in the Tonic-Clonic Seizure Model in Mice
Source: Int J Mol Sci. 2021 May 24;22(11):5537. doi: 10.3390/ijms22115537 (PMC8197343; doi:10.3390/ijms22115537)

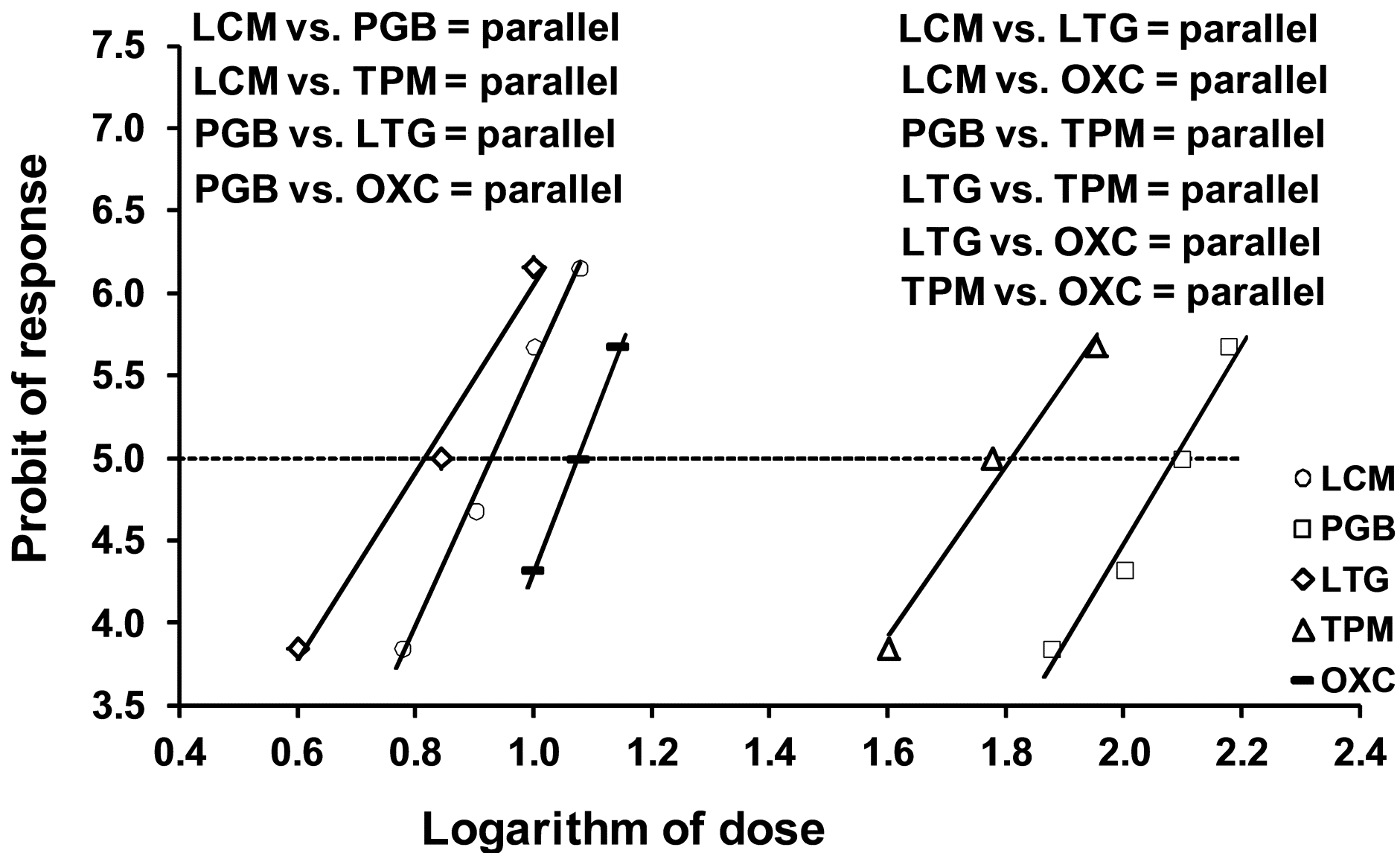

LCM ( $y = 7.886 x - 2.325$ ),  $R^2 = 0.991$ ;  $ED_{50} = 8.49 \pm 0.88$  mg/kg  
 PGB ( $y = 6.038 x - 7.586$ ),  $R^2 = 0.968$ ;  $ED_{50} = 121.52 \pm 13.36$  mg/kg  
 LTG ( $y = 5.688 x + 0.360$ ),  $R^2 = 0.984$ ;  $ED_{50} = 6.54 \pm 1.32$  mg/kg  
 TPM ( $y = 5.181 x - 4.372$ ),  $R^2 = 0.978$ ;  $ED_{50} = 64.38 \pm 10.10$  mg/kg  
 OXC ( $y = 9.210 x - 4.902$ ),  $R^2 = 0.998$ ;  $ED_{50} = 11.89 \pm 0.86$  mg/kg

Supplement: Supplementary file 1 [file ijms-22-05537-s001.zip › ijms-1209987-Figure S1.pdf]
